# IgG4-related sialadenitis: IgG4 is helpful, but biopsies are still crucial

**DOI:** 10.1186/s13075-015-0888-7

**Published:** 2015-12-18

**Authors:** Torsten Witte, Hendrik Schulze-Koops

**Affiliations:** Klinik für Immunologie und Rheumatologie, Carl-Neuberg-Straße 1, 30625 Hannover, Germany; Sektion Rheumatologie und Klinische Immunologie, Medizinische Klinik und Poliklinik IV, Ludwig-Maximilians-Universität München, Pettenkoferstraße 8a, 80336 München, Germany

## Abstract

IgG4-related disease is rare, but a frequent differential diagnosis for malignant and for autoimmune diseases. Li and colleagues report the largest cohort of patients with IgG4-related sialadenitis. The observations reveal that the most important diagnostic step is obtaining biopsies. In addition, the IgG4 serum concentration may be a biomarker for the disease progression.

In the article by W. Li and colleagues [1] a large cohort of patients with IgG4-related sialadenitis, which is a frequent manifestation of IgG4-related disease (IgG4-RD), is described. IgG4-RD was discovered in 2001, when an association of type I autoimmune pancreatitis with an elevation of serum IgG4 was published [2]. Subsequently, further disorders sharing this association were characterized and in 2010 the concept of an IgG4-RD was proposed for disorders sharing the features of lymphoplasmacytic tissue infiltration, predominantly of IgG4^+^ plasma cells, storiform fibrosis, and obliterative phlebitis [3]. Meanwhile, diagnostic criteria for IgG4-RD have been developed [4]. IgG4-RD now comprises a broad spectrum of clinical manifestations, including chronic sclerosing or IgG4-related sialadenitis and its variants Mikulicz disease (lacrimal, parotid, and submandibular gland enlargement) and Küttner’s tumor (isolated submandibular gland enlargement). Even though IgG4-RD including IgG4-related sialadenitis is rare, it is a frequent differential diagnosis for malignant diseases and for autoimmune diseases; in the case of IgG4-related sialadenitis in particular for Sjögren’s syndrome. However, there are still several open questions:

1. Is IgG4-RD an autoimmune disease or an allergic disease?
2. Are the IgG4 antibodies pathogenic, or just a marker for a strong T-helper type 2 polarization of IgG4-RD?
3. Is IgG4-RD a single disease entity at all, or a mixture of various diseases with a similar pathogenesis?

Within IgG4-RD, at least two major subgroups appear to be present: a subgroup with only head or neck involvement, which affects females and males equally; and a subgroup with either systemic involvement of many organs or of one organ outside the head and neck region, which affects mostly males (80 %) [5]. Both forms may coincide.

To answer these questions on IgG4-RD, an exact diagnosis and therefore optimization of the diagnostic procedures is crucial. We thank Li et al. [1] for their effort to compare the association of various clinical signs, laboratory parameters, computed tomography (CT) scans, and histopathology with IgG4-related sialadenitis, which has to be distinguished from other causes of glandular enlargement such as lymphoma or Sjögren’s syndrome.

A large number of patients with IgG4-related sialadenitis (*n* = 42) were carefully analyzed. Confirming earlier studies and different from patients with the systemic subgroup of IgG4-RD involving abdominal organs, females were more frequently involved than males. Concomitant autoimmune pancreatitis was observed in only 9 % of the patients, but the majority of the patients had a history of allergies. CT scans were more sensitive than the clinical examination in detection of enlarged salivary glands. In our opinion, ultrasound and magnetic resonance imaging (MRI) should be compared with CT scans in subsequent studies, since these techniques avoid radiation exposure, ultrasound would be easier to obtain, and MRI is currently the standard in assessing glandular structure.

Cervical lymphadenopathy was found in 71 % of the patients and should therefore raise the suspicion of IgG4-RD in patients with enlarged salivary glands.

In the laboratory workup, elevated IgG4 was the most important parameter and was found in 95 % of the patients. This frequency is surprisingly high in view of studies on other manifestations of IgG4-RD, in which only approximately 60 % of the patients had elevated IgG4. This suggests that either IgG4-related sialadenitis differs from other manifestations of IgG4-RD in terms of the production of IgG4, or that there may have been a selection bias. Elevated serum IgE and blood eosinophilia (in 79 % and in 20 % of the patients, respectively) are helpful parameters in raising the suspicion of IgG4-RD, but similar to elevated IgG4 do not differentiate from malignancies. The most important diagnostic procedure is therefore a biopsy, which should be taken from a major salivary gland, preferably from the submandibular glands. In immunohistochemistry not only the previously defined features of IgG4-RD such as obliterative phlebitis or storiform fibrosis were identified, but also eosinophilia, present in 47 % of the patients, was helpful. Most important, biopsies can distinguish IgG4-related sialadenitis from malignancies of the glands, confirm the diagnosis, and justify the subsequent prolonged corticosteroid therapy.

The findings of Li et al.’s study help to distinguish IgG4-related sialadenitis from Sjögren’s syndrome, which historically were considered to be related or even identical diseases. IgG4-related sialadenitis more often affects males and manifests as diffuse gland swelling rather than localized involvement of the parotid glands, the IgG4 is elevated, the presence of SS-A antibodies is rare, and histologically the plasma cells infiltrating the glands express IgG4 and the fibrosis is more pronounced as in Sjögren’s syndrome and storiform.

This study by Li et al. is the largest so far with regard to histological analysis of the glands of patients with IgG4-related sialadenitis. Interestingly, the IgG4 serum concentration correlated with the glandular fibrosis and the reduction of saliva production and could therefore be a future biomarker for the disease progression. This is a new finding, which may be important in monitoring the efficacy of treatment of IgG4-related sialadenitis.

Not all of the open questions on IgG4-RD can be answered by this study. The data give us information on diagnostic procedures and stress the importance of obtaining biopsies. The fact that most of the patients have an elevated IgE, a history of allergies, and eosinophilic infiltrates in the affected glands suggests an influence of interleukin-4 in the pathogenesis but does not yet prove an allergic rather than an autoimmune etiology of the disorder.

## Abbreviations

CT: Computed tomography
IgG4-RD: IgG4-related disease
MRI: Magnetic resonance imaging
